# Genome sequence of the chromate-resistant bacterium *Leucobacter salsicius* type strain M1-8^T^

**DOI:** 10.4056/sigs.4708537

**Published:** 2013-12-31

**Authors:** Ji-Hyun Yun, Yong-Joon Cho, Jongsik Chun, Dong-Wook Hyun, Jin-Woo Bae

**Affiliations:** 1 Department of Life and Nanopharmaceutical Sciences and Department of Biology, Kyung Hee University, Seoul, South Korea; 2 ChunLab Inc., Seoul National University, Seoul, South Korea

**Keywords:** chromate resistance, *Leucobacter salsicius*, *Microbacteriaceae*

## Abstract

*Leucobacter salsicius* M1-8^T^ is a member of the *Microbacteriaceae* family within the class *Actinomycetales*. This strain is a Gram-positive, rod-shaped bacterium and was previously isolated from a Korean fermented food. Most members of the genus *Leucobacter* are chromate-resistant and this feature could be exploited in biotechnological applications. However, the genus *Leucobacter* is poorly characterized at the genome level, despite its potential importance. Thus, the present study determined the features of *Leucobacter salsicius* M1-8^T^, as well as its genome sequence and annotation. The genome comprised 3,185,418 bp with a G+C content of 64.5%, which included 2,865 protein-coding genes and 68 RNA genes. This strain possessed two predicted genes associated with chromate resistance, which might facilitate its growth in heavy metal-rich environments.

## Introduction

The strain M1-8^T^ (= KACC 21127^T^ = JCM 16362^T^) is the type strain of the species *Leucobacter salsicius* [1], which was isolated from a Korean salt-fermented seafood known as “jeotgal” in Korean. The species epithet was derived from the Latin word *salsicius*, which means salty [1]. The genus *Leucobacter* was proposed in 1996 [2] and comprises a group of related Gram-positive, aerobic, non-motile, rod-shaped bacteria. *Leucobacter* strains have been recovered from a variety of ecological niches, including activated sludge from soil [3], wastewater [4-6], river sediments containing chromium [5], nematodes [7,8], food [1,9], potato plant phyllosphere [10], chironomid egg masses [11], air [12], soil [13], and feces [14]. Several *Leucobacter* strains have been reported to possess chromate resistance [1,4,11]. At present, there are 18 validly named *Leucobacter* species, but the only sequenced genomes in this genus were *Leucobacter* sp. UCD-THU [15] and *L. chromiiresistens* [16]. Among them, the highest resistance to chromate (up to 300 mM K_2_CrO_4_) was observed in *L. chromiiresistens*, *in vivo* [13]. However, no information has been generated on genes related to the mechanism of chromate resistance .

*L. salsicius* strain M1-8^T^ has lower chromate resistance than *L. chromiiresistens* but it still exhibits moderate resistance (up to 10.0 mM Cr(VI)). Thus, the genomic analysis of *L. salsicius* M1-8^T^ should help us to understand the molecular basis of adaptation to a chromium-contaminated environment. The present study determined the classification and features of *Leucobacter salsicius* strain M1-8^T^, as well as its genome sequence and gene annotations.

## Classification and features

### 16S rRNA analysis

A representative genomic 16S rRNA gene of strain M1-8^T^ was compared with those obtained using NCBI BLAST [17] with the default settings (only highly similar sequences). The most frequently occurring genera were *Leucobacter* (65.0%), unidentified bacteria (20.0%), *Curtobacterium* (6.0%), *Microbacterium* (5.0%), *Leifsonia* (2.0%), *Subtercola* (1.0%), and *Zimmermannella* (1.0%) (100 hits in total). The species with the Max score was *Leucobacter exalbidus* (AB514037), which had a shared identity of 99.0%.

**Figure 1:**
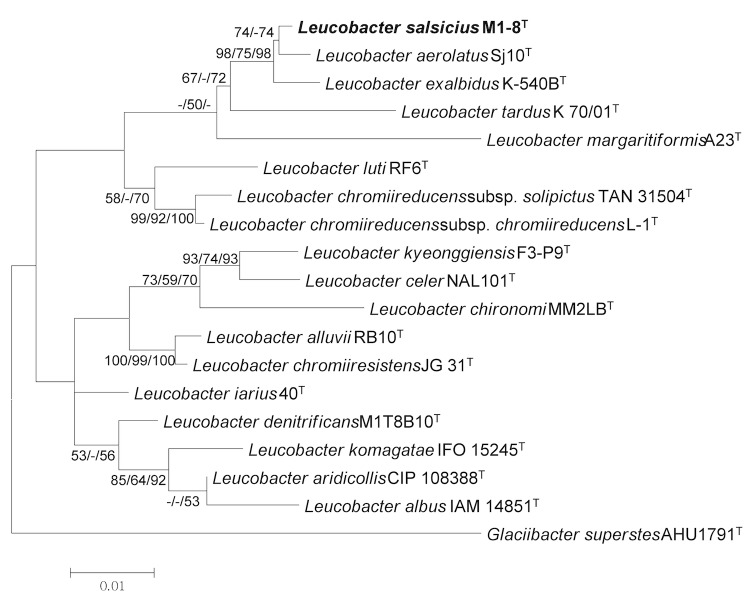
Phylogenetic tree showing the position of *Leucobacter salsicius* relative to the type strains of other species within the genus *Leucobacter*, using *Glaciibacter superstes* AHU1791^T^ as the outgroup. The sequences were aligned using CLUSTALW [18] and the phylogenetic tree was inferred from 1,390 aligned characteristics of the 16S rRNA gene sequence using the maximum-likelihood (ML) algorithm [20] with MEGA5 [19]. The branches are scaled in terms of the expected number of substitutions per site. The numbers adjacent to the branches are the support values based on 1,000 ML bootstrap replicates [20] (left), 1,000 maximum-parsimony bootstrap replicates [21] (middle), and 1,000 neighbor-joining bootstrap replicates [22] (right), for values >50%.

The multiple sequence alignment program CLUSTALW [18] was used to align the 16S rRNA gene sequences from M1-8^T^ and related taxa. Phylogenetic trees were constructed based on the aligned gene sequences using the maximum-likelihood, maximum-parsimony, and neighbor-joining methods based on 1,000 randomly selected bootstrap replicates using MEGA version 5 [19]. Strain M1-8^T^ shared 99.1% nucleotide sequence similarity with *L. aerolatus* Sj10^T^, the closest validated *Leucobacter* species according to the phylogeny (Figure 1). Figure 1 shows the phylogenetic position of *L. salsicius* in the 16S rRNA-based tree. The sequence of the single 16S rRNA gene copy found in the genome did not differ from the previously published 16S rRNA sequence (GQ352403).

### Morphology and physiology

**Figure 2:**
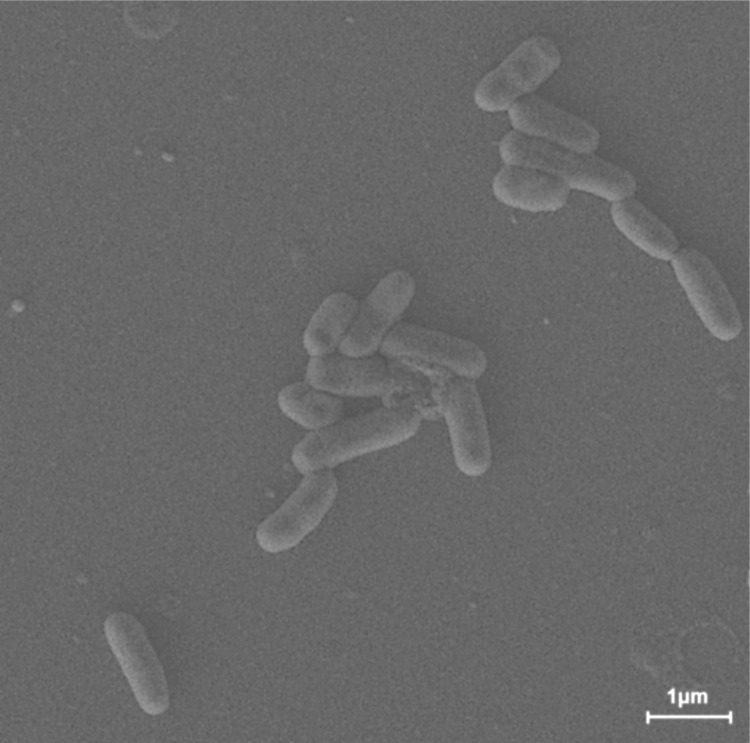
Scanning electron micrograph of *Leucobacter salsicius* M1-8^T^, which was obtained using a SUPRA VP55 (Carl Zeiss) at an operating voltage of 15 kV. The scale bar represents 1 μm.

Strain M1-8^T^ is classified as class *Actinobacteria*, order *Actinomycetales*, family *Microbacteriaceae*, genus *Leucobacter* (Table 1) [1]. The strain *L. salsicius* M1-8^T^ was isolated from a Korean salt-fermented food that contains tiny shrimp (shrimp jeotgal). The cells of strain M1-8^T^ were rod-shaped, 1.0–1.5 μm in length, and 0.4–0.5 μm in diameter (Figure 2). No flagella were observed. The colonies were cream in color and circular with entire margins on marine agar medium. Strain M1-8^T^ was aerobic and Gram-positive (Table 1). Optimum growth was observed at 25–30°C, at pH 7.0–8.0, and in the presence of 0–4% (w/v) NaCl. The tolerance of Cr (VI) was observed at up to 10.0 mM K_2_CrO_4_. The physiological characteristics, such as the growth substrates of M1-8^T^, were described in detail in a previous study [1].

**Table 1:** Classification and general features of *L. salsicius* M1-8^T^ according to the Minimum Information about a Genome Sequence (MIGS) recommendations [23]

**MIGS ID**	**Property**	**Term**	**Evidence code^a^**
	Current classification	Domain *Bacteria*	TAS [24]
		Phylum *Actinobacteria*	TAS [25]
		Class *Actinobacteria*	TAS [26]
		Order *Actinomycetales*	TAS [26-29]
		Family *Microbacteriaceae*	TAS [26,27,30,31]
		Genus *Leucobacter*	TAS [2]
		Species *Leucobacter salsicius*	TAS [1]
		Type strain M1-8^T^	TAS [1]
	Gram stain	Positive	TAS [1]
	Cell shape	Rod-shaped	TAS [1]
	Motility	Non-motile	TAS [1]
	Sporulation	Not reported	
	Temperature range	Mesophile	TAS [1]
	Optimum temperature	25–30°C	TAS [1]
	pH	pH 7–8	TAS [1]
MIGS-22	Oxygen requirement	Aerobic	TAS [1]
	Carbon source	Heterotroph	TAS [1]
	Energy metabolism	Not reported	
MIGS-6	Habitat	Fermented food	TAS [1]
MIGS-6.3	Salinity	Halotolerant	TAS [1]
MIGS-15	Biotic relationship	Free-living	NAS
MIGS-14	Pathogenicity	Not reported	NAS
	Isolation	Fermented food (Shrimp jeotgal, a Korean salt-fermented food)	TAS [1]
MIGS-4	Geographic location	South Korea	TAS [1]
MIGS-5	Sample collection date	May 2009	NAS
MIGS-4.1	Latitude	Not reported	
MIGS-4.1	Longitude	Not reported	
MIGS-4.3	Depth	Not reported	
MIGS-4.4	Altitude	Not reported	

The evidence codes are as follows. TAS: traceable author statement (i.e., a direct report exists in the literature). NAS: non-traceable author statement (i.e., not observed directly in a living, isolated sample, but based on a generally accepted property of the species, or anecdotal evidence). These evidence codes are derived from the Gene Ontology project [32].

### Chemotaxonomy

The peptidoglycan hydrolysate from strain M1-8^T^ contained alanine, 2,4-diaminobutyric acid (DAB), γ-aminobutyric acid (GABA), glutamic acid, and glycine. The predominant fatty acids (>10% of the total) in M1-8^T^ were anteiso-C_15:0_ (63.6%), anteiso- C_17:0_ (16.7%), and iso-C_16:0_ (14.2%). The polar lipid profile of strain M1-8T contained diphosphatidylglycerol and an unknown glycolipid. The major menaquinone in M1-8^T^ was MK-11 and the minor menaquinones were MK-10 and MK-7.

## Genome sequencing and annotation

### Genome project history

*L. salsicius* strain M1-8^T^ was selected for genome sequencing based on its environmental potential and is part of the Next-Generation BioGreen 21 Program (No.PJ008208). The genome sequence was deposited in DDBJ/EMBL/GenBank under accession number AOCN00000000 and the genome project was deposited in the Genomes On Line Database [33] under Gi21829. The sequencing and annotation were performed by ChunLab Inc., South Korea. A summary of the project information and the associations with “Minimum Information about a Genome Sequence” (MIGS) [34] are shown in Table 2.

**Table 2:** Genome sequencing project information

**MIGS ID**	**Property**	**Term**
MIGS-31	Finishing quality	Improved high-quality draft
MIGS-28	Libraries used	454 PE library (8 kb insert size), Illumina PE library (150 bp)
MIGS-28.2	Number of reads	4,157,212 sequencing reads
MIGS-29	Sequencing platforms	PacBio RS, Illumina GAii, 454-GS-FLX-Titanium
MIGS-31.2	Sequencing coverage	189.78 × Illumina; 7.96 × pyrosequence; 15.88 × PacBio
MIGS-30	Assemblers	Roche gsAssembler version 2.6, CLCbio CLC Genomics Workbench version 5.0
MIGS-32	Gene-calling method	Prodigal 2.5
	INSDC ID	AOCN01000000
	GenBank Date of Release	April 3, 2013
	GOLD ID	Gi21829
	NCBI project ID	175945
	Database: IMG	2526164546
MIGS-13	Source material identifier	KACC 21127^T^, JCM 16362^T^
	Project relevance	Environmental and biotechnological

### Growth conditions and DNA isolation

*L. salsicius* strain M1-8^T^ was cultured aerobically in marine agar medium at 30°C. Genomic DNA was extracted using a G-spin DNA extraction kit (iNtRON Biotechnology), according to the standard protocol recommended by the manufacturer.

### Genome sequencing and assembly

The genome was sequenced using a combination of an Illumina Hiseq system with a 150 base pair (bp) paired-end library, a 454 Genome Sequencer FLX Titanium system (Roche) with an 8 kb paired-end library, and a PacBio RS system (Pacific Biosciences). The Illumina reads were assembled using CLC Genomics Workbench ver. 5.0. The initial assembly was converted for the CLC Genomics Workbench by constructing fake reads from the consensus to collect the read pairs in the Illumina paired-end library. The 454 paired-end reads were assembled with Illumina data using gsAssembler ver. 2.6 (Roche) and the PacBio sequences were clustered into overlapping assembled data. CodonCode Aligner and CLC Genomics Workbench 5.0 were used for sequence assembly and quality assessment in the subsequent finishing process. The Illumina (189.78-fold coverage; 4,003,590 reads), PacBio (88-fold coverage; 23,441 reads), and 454 sequencing (7.96-fold coverage; 130,181 reads) platforms provided 213.62 × coverage (total 4,157,212 sequencing reads) of the genome. The final assembly identified one scaffold that included 28 contigs.

### Genome annotation

The genes in the assembled genome were predicted using Integrated Microbial Genomes - Expert Review (IMG-ER) platform as part of the DOE-JGI genome annotation pipeline [35], followed by a round of manual curation using the JGI GenePRIMP pipeline. Comparisons of the predicted ORFs using the SEED [36], NCBI COG [37], Ez-Taxon-e [38], and Pfam [39] databases were conducted during gene annotation. Additional gene prediction analyses and functional annotation were performed with the Rapid Annotation using Subsystem Technology (RAST) server databases [40] and the gene-caller GLIMMER 3.02. RNAmer 1.2 [41] and tRNAscan-SE 1.23 [42] were used to identify rRNA genes and tRNA genes, respectively. The CLgenomics^TM^ 1.06 (ChunLab) was used to visualize the genomic features.

## Genome properties

**Figure 3:**
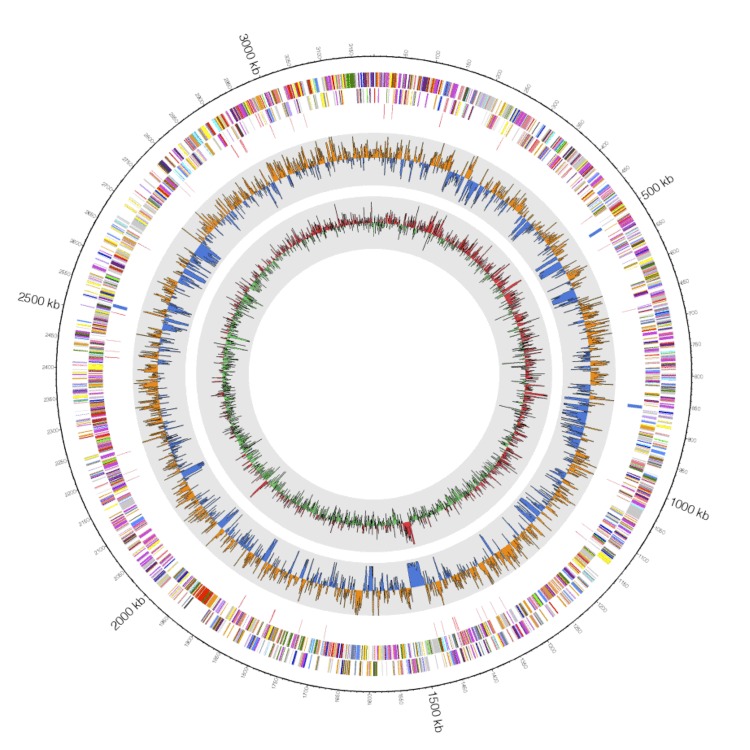
Graphical map of the largest scaffold. From the outside to the center: genes on the reverse strand (colored according to the COGs categories), genes on the forward strand (colored according to the COGs categories), and RNA genes (tRNAs in red and rRNAs in blue). The inner circle shows the GC skew, where yellow indicates positive values and blue indicates negative values. The GC ratio is shown in red/green, which indicates positive/negative, respectively.

The genome comprised a circular chromosome with a length of 3,185,418 bp and a G+C content of 64.5% (Figure 3 and Table 3). Of the 2,933 predicted genes, 2,865 were protein-coding genes and 68 were RNA genes (three 5S rRNA genes, three 16S rRNA genes, three 23S rRNA genes, 51 predicted tRNA genes, and eight miscRNA genes). The majority of the protein-coding genes (2,275 genes; 77.6%) was assigned putative functions, while the remainder was annotated as hypothetical proteins (182 genes). The genome properties and statistics are summarized in Table 3. The distributions of genes among the COGs functional categories are shown in Table 4.

**Table 3:** Genome statistics

**Attribute**	**Value**	**% of total^a^**
Genome size (bp)	3,185,418	100
DNA coding region (bp)	2,905,046	91.20
DNA G+C content (bp)	2,054,445	64.5
Total genes	2,933	100
RNA genes	68	2.32
rRNA operons	3	0.31
Protein-coding genes	2,865	97.68
Genes with predicted functions	2,275	77.57
Genes in paralog clusters	2,357	80.36
Genes assigned to COGs	2,210	75.35
Genes assigned Pfam domains	2331	79.47
Genes with signal peptides	195	6.65
Genes with transmembrane helices	784	26.73

^a^The totals are based on either the size of the genome in base pairs or the total number of protein-coding genes in the annotated genome.

**Table 4:** Number of genes associated with general COGs functional categories

**Code**	**Value**	**% age^a^**	**Description**
J	156	6.38	Translation, ribosomal structure, and biogenesis
A	4	0.16	RNA processing and modification
K	218	8.91	Transcription
L	167	6.83	Replication, recombination, and repair
B	1	0.04	Chromatin structure and dynamics
D	21	0.86	Cell cycle control, cell division, and chromosome partitioning
Y	0	0.00	Nuclear structure
V	40	1.64	Defense mechanisms
T	100	4.09	Signal transduction mechanisms
M	112	4.58	Cell wall/membrane/envelope biogenesis
N	0	0.00	Cell motility
Z	1	0.04	Cytoskeleton
W	0	0.00	Extracellular structures
U	32	1.31	Intracellular trafficking, secretion, and vesicular transport
O	69	2.82	Posttranslational modification, protein turnover, and chaperones
C	131	5.36	Energy production and conversion
G	129	5.27	Carbohydrate transport and metabolism
E	315	12.88	Amino acid transport and metabolism
F	74	3.03	Nucleotide transport and metabolism
H	101	4.13	Coenzyme transport and metabolism
I	81	3.31	Lipid transport and metabolism
P	154	6.30	Inorganic ion transport and metabolism
Q	51	2.09	Secondary metabolites biosynthesis, transport, and catabolism
R	307	12.55	General function prediction only
S	182	7.42	Function unknown
-	723	24.65	Not in COGs

^a^The total is based on the total number of protein-coding genes in the annotated genome.

## Insights from the genome sequence

*Leucobacter salsicius* M1-8^T^ and *Leucobacter* members, such as *L. chromiireducens*, *L. aridicollis*, *L. luti*, and *L. alluvii*, have been shown to possess chromate resistance in previous studies, while Zhu et al. reported the reduction of chromate by *Leucobacter* sp. [43]. In the present study, the genome analysis of *Leucobacter salsicius* M1-8^T^ detected two copies of chromate transport protein A (ChrA), which is a membrane protein that confers heavy metal tolerance via chromate ion efflux from the cytoplasm. Potentially, this gene is a key feature that allows *Leucobacter* to adapt to chromate-contaminated environments. The genome sequence of *L. salsicius* M1-8^T^ should provide deeper insights into the molecular mechanisms that underlie chromium tolerance and it may facilitate the development of biotechnological applications to improve chromium-contaminated field sites.
